# Research Landscape of Artificial Intelligence and e-Learning: A Bibliometric Research

**DOI:** 10.3389/fpsyg.2022.795039

**Published:** 2022-02-16

**Authors:** Kan Jia, Penghui Wang, Yang Li, Zezhou Chen, Xinyue Jiang, Chien-Liang Lin, Tachia Chin

**Affiliations:** ^1^School of Management, Zhejiang University of Technology, Hangzhou, China; ^2^School of Cultural Creativity and Management, Communication University of Zhejiang, Hangzhou, China; ^3^School of Economics, Zhejiang University of Technology, Hangzhou, China; ^4^College of Science and Technology Ningbo University, Ningbo, China

**Keywords:** artificial intelligence, online learning, technological education, bibliometrics research, Web of Science Publications

## Abstract

While an increasing number of organizations have introduced artificial intelligence as an important facilitating tool for learning online, the application of artificial intelligence in e-learning has become a hot topic for research in recent years. Over the past few decades, the importance of online learning has also been a concern in many fields, such as technological education, STEAM, AR/VR apps, online learning, amongst others. To effectively explore research trends in this area, the current state of online learning should be understood. Systematic bibliometric analysis can address this problem by providing information on publishing trends and their relevance in various topics. In this study, the literary application of artificial intelligence combined with online learning from 2010 to 2021 was analyzed. In total, 64 articles were collected to analyze the most productive countries, universities, authors, journals and publications in the field of artificial intelligence combined with online learning using VOSviewer through WOS data collection. In addition, the mapping of co-citation and co-occurrence was explored by analyzing a knowledge map. The main objective of this study is to provide an overview of the trends and pathways in artificial intelligence and online learning to help researchers understand global trends and future research directions.

## Introduction

The integration of technology into teaching has become an important part of the educational environment in recent years (Martins and Kellermanns, 2004; Teo, 2016; Huang et al., 2020). The steady development of online learning not only promotes the transfer of curriculum forms from offline to online learning (Lin et al., 2021; Wang et al., 2021) but also urges universities and teachers to use various online learning technologies to assist teaching (Mulder, 2013). For instance, the integration of wireless networks, sensing and mobile technologies into the field of education brings innovative changes to education and learning (Tang et al., 2021) and forms the concept of e-learning. Compared to traditional face-to-face education, online learning has more advantages (Piccoli et al., 2001). For example, the liberating interactions between learners and instructors or learners and learners have been facilitated, and the limitations of time and space in the asynchronous and synchronous learning network model have been reduced (Trentin, 1997; Katz, 2000, 2002).

To better enable the e-learning system to provide personalized or appropriate learning content, learning guidance, learning feedback, learning paths or interfaces (Hwang, 2014; Rastegarmoghadam and Ziarati, 2017; Conde et al., 2020). As an emerging technology, artificial intelligence has been extensively explored worldwide in the past few decades, and the application of artificial intelligence in e-learning (AIEL) has also been a serious issue (Tang et al., 2021), especially its application in many disciplines. For example, Hwang and Tu (2021) conducted a literature review on the application of artificial intelligence in mathematics education. Artificial intelligence enables students to interact with virtual patients and obtain the diagnostic information and feedback of specific patients in the field of medical education (Khumrin et al., 2017). The neural network model of artificial intelligence can also be used in science education (Iyanda et al., 2018). In addition, researchers have also studied the application of artificial intelligence in online learning from other perspectives. García et al. (2007), for example, used artificial intelligence to identify the heterogeneity of students’ learning styles. Osmanbegovic and Suljic (2012) used data mining methods to predict students’ performances. Some scholars have also used new algorithms (Kurilovas et al., 2015) and models (Ben Ammar et al., 2010; Caputi and Garrido, 2015) to develop learning systems.

Presently, there are also relevant literature reviews in the AIEL field, but some of these literature reviews only focus on a certain discipline (e.g., mathematics, Hwang and Tu, 2021) or a specific field (Chan-Olmsted, 2019; George and Lal, 2019). Tang et al. (2021) conducted a systematic review and co-cited network analysis on the application trend of artificial intelligence in online learning. However, the COVID-19 pandemic in early 2020 accelerated the change in the performance mode of physical teaching and blended learning (Jin et al., 2021; Lin et al., 2021; Mo et al., 2021). Although some studies have discussed the application of artificial intelligence in online learning, they only focused on co-citation network analysis (Hwang and Tu, 2021; Tang et al., 2021) and did not discuss the application development of artificial intelligence in teaching at different time points and the application trend of artificial intelligence in online learning during the pandemic. Furthermore, they did not explore the possible development trend of artificial intelligence applications in online learning after the pandemic.

Therefore, considering the above discussion, the previous research on AIEL application from 2010 to 2021 was analyzed and extracted in this study. Admittedly, the reason for choosing 2010 as the main research target is because Hinton’s team used the Alex Net, a convolutional neural network, to win the ImageNet image recognition competition in 2012, proving the potential of deep learning to the world and attracting the attention of the academic world (Hinton et al., 2012; Krizhevsky et al., 2012). It was also from this moment that research on artificial intelligence entered a period of explosion, which urged many research fields, such as the field of education, to emerge with high-frequency knowledge association related to artificial intelligence. Therefore, by applying the literature metrology method, the literature during the selected period was analyzed to extract key information, such as periodicals, authors, institutions, countries, years, keywords and references in academic publications, thus forming knowledge network maps and providing references for the related researchers and practitioners. Specifically, the research questions (RQ) raised in this study are as follows:

RQ1: Who are the main authors published in the field of AIEL and what are their institutional and county affiliations?
RQ2: What kinds of major journals and keywords are used in the field of AIEL and what are the connections and differences between them?
RQ3: What is the co-citation of the AIEL literature? What were the most frequently cross-referenced research streams in this field during the selected period? What is the visualized structure of the main AIEL literature from the perspective of these papers?

This research is organized as follows: Section “Literature Review” describes the literature review of this study. Section “Research Methodology” presents the research methodology. Section “Results” presents the data analysis and results, and section “Discussion and Conclusion” highlights the discussion and conclusion.

## Literature Review

### Artificial Intelligence

Artificial intelligence is one of the branches of computer science and is defined as “the theory and development of computer systems capable of performing tasks that normally require human intelligence” (Miyazawa, 2019). The field of artificial intelligence research is defined as the study of “intelligent agents” and any device that can sense its surrounding environment and act to maximize its chances of success at a certain goal (Song and Wang, 2020). Studies have interpreted artificial intelligence as a system of rational thinking, rational behavior, or both (Kok et al., 2009).

Many related technologies have been integrated into artificial intelligence to simulate human thought processes and intelligent behavior, such as neural networks, expert systems, deep learning, symbolic machine learning, speech recognition, image recognition, natural language processing and statistical analysis, or others that can be classified as artificial intelligence technologies (Lu and Yang, 2018). Artificial intelligence has made considerable progress recently and has been extensively applied in various fields around the world, bringing outstanding value and possessing great potential, such as practical application in medical-related fields (He et al., 2019), augmented online language learning (Li and Wang, 2020), scientific research based on the neural network model (Iyanda et al., 2018), personality and affective differences in the psychology sector (Latham et al., 2012) and basic discipline education in mathematical science (Kok et al., 2009; Colchester et al., 2017).

Since 2012, artificial intelligence research has been expanding (Hinton et al., 2012; Krizhevsky et al., 2012). Due to its advantages over geography and time constraints, this technology is considered a necessary resource for big data analysis (Song et al., 2020). Particularly, in the field of education, a high-frequency artificial intelligence–related knowledge association has emerged, aiming to improve the efficiency of educational communication and to provide an assessment teaching system with distinct individual differences (Tang et al., 2021). Therefore, a large-scale application of artificial intelligence combined with teaching has emerged, providing a comprehensive learning platform with a coherent learning sequence and formative assessment (Vílchez-Román et al., 2020). Based on data mining technology (Hu et al., 2014), an early warning prediction of students’ academies is conducted. Further, based on the holistic multi-dimensional instructional design model, an adaptive learning environment for dynamic curriculum design is realized, and based on ontology and sequential pattern mining, the problem of cold starts and sparse ratings is solved to generate a knowledge recommendation system with final suggestions for target learners (Tarus et al., 2017). Artificial intelligence, combined with online learning, enables students with different knowledge levels, personalities and emotions to customize education courses. Simultaneously, it has proved useful in the areas of employee training, knowledge updating, professional development and skills training (Kavitha and Lohani, 2019).

### Bibliometric Analysis Using Knowledge Mapping

Knowledge mapping is a new development of scientometrics and infometrics that is based on science and involves the interdisciplinary application of applied mathematics, information science and computer science Chen, 2006, 2017). In recent years, an increasing number of researchers have been using various knowledge mapping tools to analyze the development trends and evolution processes of several disciplines. CiteSpace and VOSviewer are two major visualization tools in bibliometric research and other graphic science or scientific discipline research, both of which can import data directly from Web of Science and other bibliometric databases to generate visualizations (Song et al., 2020). Comparing the differences between the two, VOSviewer is more accurate in clustering algorithms, while CiteSpace is better in presenting evolution and has the advantages of beautiful images and easier interpretation. Therefore, this study uses VOSviewer software to analyze citation, co-citation and the most commonly used author keywords in articles (Hwang and Tu, 2021). VOSviewer is a computer program that can construct and view bibliometric maps of bibliomapies.

The software, constructed by van Eck and Waltman (2010), is mainly used to construct literature maps and co-author relationships and to conduct co-citation analysis and literature coupling analysis. Different from most computer programs used for bibliometrics, VOSviewer pays particular attention to the graphical representation of bibliometrics and plays a prominent role in the field of large bibliometric maps due to its easy interpretation (van Eck and Waltman, 2010). More importantly, VOSviewer can conduct text mining and can construct a network of important terms in literature (Flis and van Eck, 2018), particularly playing an irreplaceable role in exploring literature reviews according to time sequence. Thus, it has been extensively used in bibliometric reviews in various fields over the past decades: research of scientific software by a link-based approach (Orduna-Malea and Costas, 2021), visual analysis of Catholic health maintenance studies (Ivanitskaya et al., 2021), the development trend of e-waste research (Corsini et al., 2012), development of European political science (Mas-Verdu et al., 2021), trend analysis of financial inclusion (Gálvez-Sánchez et al., 2021), exploration of the decision-making process of higher education institutions using visual analysis technology (Vílchez-Román et al., 2020) and trend analysis of library and information science (Song et al., 2020). Based on the results of previous studies, it can be understood that most studies use the VOSviewer tool to explore relevant issues in co-citation and coupling analyzes in the literature (see Table 1).

**TABLE 1:** Research topics on bibliometric research.

**Author (Year)**	**Major Content**	**Research Area**
Van Eck and Waltman (2017)	Provide clustered and analyzed solutions in the bibliometrics of scientific publications.	Scientific Publications Cluster
Donthu et al. (2020)	Analyze the influence, prominent themes and the most prolific authors, institutes and countries of JBR magazine and divide the JBR publications into six clusters to describe co-authors, bibliomapic coupling of authors and their institutes and countries.	Journal of Business Research
Zou et al. (2018)	Intuitively explore the knowledge base, subject distribution, research frontier and research trend of road safety research.	Road safety research mapping
Sarkodie and Strezov (2019)	Conduct the textual corpus modeling to prove the research of heterogeneity at the turning point of EKC hypothesis, which is beneficial to environmental policy making.	Environmental Kuznets curve hypothesis
Liao et al. (2018)	Explore the current situation and trend of medical big data to help medical professionals understand the advanced level of MBD and to improve the research and application of visualization methods.	Medical big data research
Colares et al. (2020)	Reveal the relationship between macroscopic plants and other pollutant removal mechanisms to improve the treatment efficiency of the FTW system.	Floating treated wetland
Ferasso et al. (2020)	Explore the relevance of a circular economy and business models in current business and management literature to highlight emerging themes, such as those related to management, supply side, demand side, network, performance, and circular business models.	Circular economy business model
Moral Muñoz et al. (2020)	Conduct an up-to-date review of various tools in bibliometric analysis based on experimental data, including data acquisition, performance analysis and sources of visualization tools.	Software tools for bibliometric analysis
Hallinger and Kovačević (2019)	Describe the paradigm shift from “school management” to “school leadership” and “student learning and development leadership,” which are identified as the “cognitive anchor” of the intelligence structure in the EA knowledge base.	Bibliomapy of educational management research
Duque-Acevedo et al. (2020)	Analyze the identification, evolution, methods and trends in the use and transformation of agricultural wastes to develop newer and better agricultural waste recycling technologies, ensure resource efficiency, sustainable production, and consumption, and reduce negative environmental impact.	Agricultural waste
Zhao et al. (2020)	Analyze the trend changes of public attention to COVID-19 and the changes and differences in hot topics of public concern at each stage.	The public in China is concerned about the COVID-19 epidemic on social media
Sweileh (2017)	Watch for two publication peaks and observe ongoing vaccine research.	Eight emerging pathogens
Gonçalves et al. (2019)	Effectively assess trends in the literature on enzyme immobilization to help fund future decisions in this area of science.	Research trend of enzyme immobilization
Faust et al. (2018)	Expound the applications of various deep learning algorithms that will be used in more healthcare areas in the future to improve diagnostic quality.	Deep learning for healthcare applications based on physiological signals

## Research Methodology

### Data Set

**FIGURE 1:**
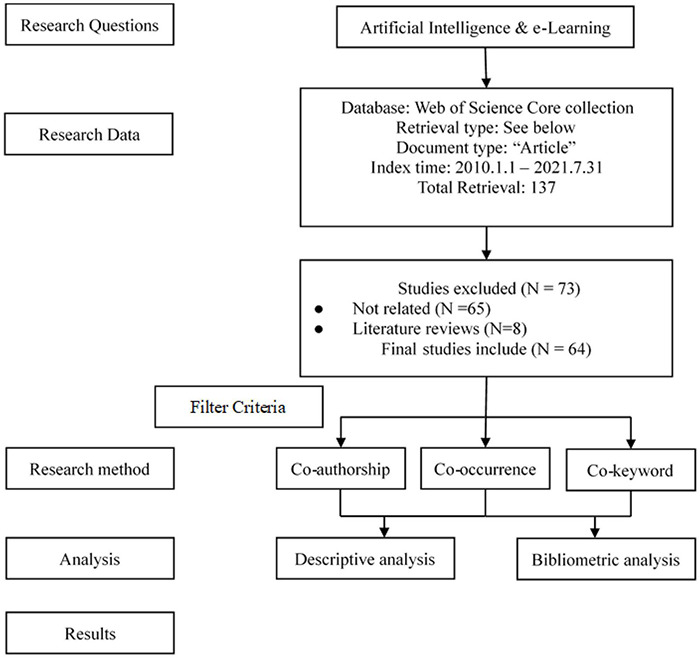
Article selection process for bibliometric mapping analysis.

All articles in this study are from the Science Citation Index (SCI) and Social Science Citation Index (SSCI) databases, obtained from the Web of Science (WOS) platform created by the Institute for Scientific Information, which provides high-quality literature data sets and is often used in scientometric research and scientific research of literature (Su et al., 2020; Vílchez-Román et al., 2020; Tang et al., 2021). Therefore, this study selected the WOS database platform as the data source for analysis. In selecting keywords, the keywords used by Zawacki-Richter et al. (2019), Hwang and Tu (2021), and Tang et al. (2021) were employed as the analysis basis of this study. To ensure the rationality of the keyword search, further discussions were conducted with three professors who have articles published in educational technology–related journals. The standards for the keyword search were determined following the suggestions of these experts in the related fields. Subsequently, according to the determined combinations of the keywords, we searched publications included in “SSCI” and “SCI” from the Web of Science database using two keywords on 31 July 2021—whose retrieval format is TS = [(“Artificial Intelligence” OR “machine Intelligence” OR “machine learning” OR “Deep learning”) AND (“e-learning” OR “Web learning” OR “Online learning”)] and retrieval standard is mainly from 1 January 2010 to 30 July 2021. Eventually, 137 papers were obtained. However, there were still inevitable problems based on the literature analysis. Some of the topics and keywords had only a “one-word description” in the papers, and the papers had no direct correlation with these keywords or topics. Therefore, subsequent data cleaning was necessary to improve the quality of samples and the credibility of the bibliometric analysis results (Jia et al., 2014). However, since it could not be done through keywords or other software, the data cleaning had to be done manually. Given that, in this study, we referred to the practice of Wang and Ngai (2020) and conducted a manual cleaning of the collected samples. In this study, two professors engaging in educational technology and information systems independently reviewed the abstract of each sample and determined, through cross-confirmation, whether the content of each sample followed the topic of this study. If the content of the sample did not follow this study’s topic, that sample would be directly deleted. Thus, the final number of the literature was 64, and the relevant basic data filtering process is shown in Figure 1.

### Co-citation Network Analysis

Co-citation is an analysis method that was first proposed by Small (1973) and refers to two pieces of literature being cited by other literature simultaneously. Citation network analysis is a combination of literature co-citation analysis and social network analysis. Core papers in the field can be identified by analyzing citation relationships between the literature (Tang et al., 2021). VOSviewer can count identical entries by comparing the reference lists of documents using.

Presently, numerous bibliometrics studies have used the citation network analysis method. For example, Liang and Liu (2018) used citation network analysis to identify core and important literature on business intelligence and big data analytics between 1990 and 2017. Through citation network analysis, Zhang et al. (2020) unearthed influential literature in the field of tourism demand prediction and analyzed the evolution process of this field according to their publication time and research methods. Wang and Ngai (2020) identified publishing trends, relevant technologies, influential publications and knowledge clusters in the field of business research methods through the main analysis of three stages: initial sample analysis, citation analysis and co-citation analysis. Through the accumulation and analysis of numerous literature on business event research methods, it has helped to transfer and accumulate useful techniques amongst disciplines and determine the direction of future research. Therefore, through co-citation network analysis, researchers can quickly discover the knowledge structure of artificial intelligence combined with online learning and the research trend in the future. For the results of the network analysis, descriptive tables and visual maps were used in this study by referring to previous practices of Kocak et al. (2019), Mou et al. (2019), and Wu et al. (2021). For the tables, frequency or publication is used to represent the weight of each node (i.e., author, institution, country, journal, and paper), while centrality is used to represent the connectivity of each node. For example, a node with high centrality indicates that the node acts as a key point linking two or more groups that display transition patterns. For the maps, the nodes show the analysis items (i.e., author, journal, reference, etc.), and the sizes of the nodes show the co-occurrence frequency of the items. The thickness and color of the nodal rings indicate the strength of an item’s co-occurrence time. The line between the nodes represents the connection relationship, the thickness of the line represents the co-citation frequency, and the color represents the strong and weak characteristics of the co-citation relationship between the nodes. Therefore, a cluster view is adopted in this study to show the evolution of knowledge over time and the collaboration between nodes.

## Results

### Publication Trends

**FIGURE 2:**
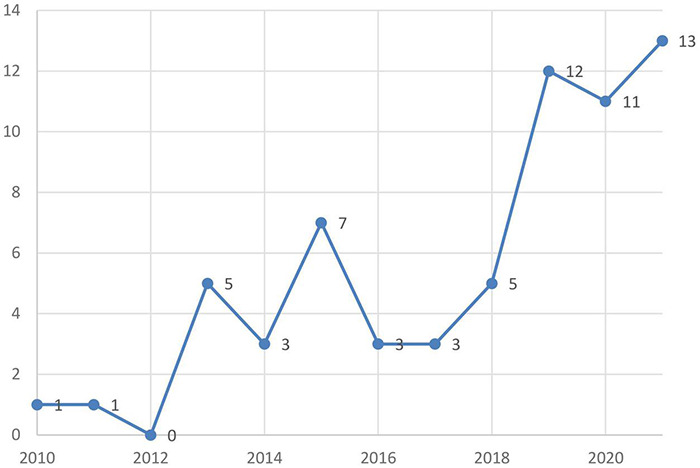
Data distribution of AIEL articles from 2010–2021.

As the development trend of a certain topic is visible from the number of papers published, it can be seen that during the period from 2010 to 2021, there was only one paper published or no relevant research output under this topic in each year, indicating that this field is in the embryonic stage. By 2013, there were five papers published in this field, which increased greatly compared to the previous 3 years. Hinton proposed the solution of deep network training in the Science Journal in 2006 (Hinton and Salakhutdinov, 2006); however, it was not until 2012 that Hinton’s team used Alex Net, a convolutional neural network, to win the ImageNet image recognition competition that it proved the potential of deep learning to the world and attracted widespread attention from the academic world (Hinton et al., 2012). It was also from this moment that the research on artificial intelligence entered a period of explosion and urged many research fields, such as the field of education, to emerge with high-frequency knowledge association related to artificial intelligence. Since then, the number of articles published in this field has remained stable and, beginning in 2019, the number of articles published in this field has increased significantly, given that the number of articles published before 2020 was only 51. Furthermore, in only the first half of 2021, the number of published papers has reached 13, which reflects the research frontier and development trend in this field (see Figure 2).

### Data Collection

Table 2 shows the journals with more than two publications under this topic, amongst which Expert Systems with Applications, Computers in Human Behavior, IEEE Access and Journal of Intelligent and Fuzzy Systems tied for first place in the number of published journals that focus on computer science, information systems, information education, psychology and communication technology. Furthermore, aiming at the literature retrieved in this study, articles published by Expert Systems with Applications were mainly gathered before 2015, and most of them are about the designs of new models or algorithms to develop new learning systems. For example, Ammar et al. (2010) is one of the first papers that qualified for retrieval, which proposed an intelligent tutoring system that uses affective computing to recognize people’s facial expressions, thus monitoring students’ behaviors while learning. In addition, this article is also the most-cited article on artificial intelligence integration teaching in this journal (80 times). In 2015, Dias et al. (2015) arranged for college professors and students to use Learning Management Systems (LMSs) in a blended learning environment for an academic year. They then explored effective learning methods through fuzzy analysis and helped the college professors and students to improve their learning efficiency. The Computers in Human Behavior journal also published four articles, and the main articles were published between 2014 and 2016. For example, Kurilovas et al. (2014, 2015) used a recommendation system to evaluate students’ learning effects to improve the modules and design of online learning courses. These two studies were cited up to 81 times. Conversely, the publications of the IEEE Access journal were mainly in 2017, and its research is mainly on applying artificial intelligence to teaching situations. For example, Barlybayev et al. (2020) proposed an intelligent system to evaluate students’ learning situations. However, other literature also explores the field of AIEL from other perspectives. Turchet et al. (2018) described and analyzed the education of music e-learning in the internet of Things. Finally, the literature retrieved from the Journal of Intelligent and Fuzzy Systems is mostly integrated into online learning from the technical level. Amongst the four journals that published the most papers on combined artificial technology and teaching, the Journal of Intelligent and Fuzzy Systems published most of these papers in 2021, showing that this journal started to include a larger number of papers on artificial intelligence and teaching from 2021. For example, Xie and Mai (2021) integrated cloud computing and artificial intelligence in MOOC platforms and applied them in the cross-cultural teaching of college English. Aiyuan and Hui (2021) proposed a model for online English learning based on artificial technology, in which students’ actual learning was analyzed through an improved deep network analysis method so that artificial intelligence could effectively improve students’ learning efficiency and demand.

**TABLE 2:** The top 10 productive journals from 2010 to 2021 (Documents ≥ 2).

**Ranking**	**Name**	**Documents**	**Citations**
1	Expert Systems with Applications	4	137
2	Computers in Human Behavior	4	120
3	IEEE Access	4	47
4	Journal of Intelligent and Fuzzy Systems	4	7
5	Education and Information Technologies	3	12
6	Applied Sciences-Basel	3	6
7	Journal of Network and Computer Applications	2	62
8	International Journal of Engineering Education	2	6
9	Sustainability	2	3
10	Educational Technology and Society	2	3

Amongst the highly cited literature, Dwivedi et al.’s (2020) research (cited 123 times) was a highly cited topic in the research and application of artificial intelligence and online learning during the epidemic, which collated the views of 12 scientists on the impact of COVID-19 on organizations and society, including information management and the use of artificial intelligence in online learning.

### Author’s Cooperation Network

Scientific research authors are the main force of scientific research institutions, representing the research development direction of a subject area. In this study, some publications of authors in the field of artificial intelligence are counted. According to the statistical results in Table 3, the distribution difference of the number of articles published between scholars is not obvious. The frequency of the highest number of articles published is two times, which is by 12 authors, and accounts for 2% of the total number of articles published. Conversely, the number of articles published once accounts for 98%. This shows that there are few high-yielding core authors in the field of artificial intelligence, and the vast majority of scholars first dabbled in the field of “artificial intelligence and online education.” Although there is no scholar with a high publication output in this field, the two papers published by Kurilovas et al. (2014, 2015) are both highly cited studies in the field of artificial intelligence and online education. However, the authors of the two papers mainly studied artificial intelligence, learning analytics and technology-enhanced learning. The academic backgrounds of the authors are mainly technology learning, information systems and other computer-related majors, suggesting that relevant research studying the combination of artificial intelligence and teaching is still directly related to the discipline and speciality of the researchers.

**TABLE 3:** Author’s cooperation network from 2010 to 2021 (Documents ≥ 2).

**Id**	**Authors**	**Research Subject**	**Documents**	**Citations**
1	Kurilovas, Eugenijus	Machine Learning, Virtual Learning Environments, ICT in Education and Technology-Enhanced Learning	2	81
2	Zilinskiene, Inga	ICT in Education, Educational Technology, Learning Analytics and Data Visualization	2	81
3	Dagiene, Valentina	Teaching Programming, Educational Technology, Mathematics Education and Technology-Enhanced Learning	2	81
4	Fatahi, Somayeh	Recommender Systems, Educational Data Mining and Technology-Enhanced Learning	2	29
5	Moradi, Hadi	Recommender Systems, Educational Data Mining and Technology-Enhanced Learning	2	29
6	Gal, Kobi	Artificial Intelligence, Human-Computer Decision Making and Learning Analytics	2	9
7	Shani, Guy	Recommender Systems, Artificial Intelligence Applied, Machine Learning and Learning Analytics	2	9
8	Baneres, David	Artificial Intelligence Applied, Machine Learning and Learning Analytics	2	4
9	Karadeniz, Abdulkadir	Artificial Intelligence Applied, Machine Learning and Learning Analytics	2	4

### Countries and Institutions

A total of 17 countries conducted research on “artificial intelligence and e-learning” between 2010 and 2021, and 10 countries published at least three articles. China, followed by Spain, is the most productive country for artificial intelligence, with 20.31% (13) of publications in the field of artificial intelligence from Chinese authors and 209 citations in other papers. It was cited 127 times in other literature, making it the most cited country (see Table 4).

**TABLE 4:** The top 10 productive countries from 2010 to 2021 (Documents ≥ 3).

**Id**	**Countries**	**Documents**	**Citations**
1	China	13	40
2	Spain	11	127
3	India	8	107
4	United States	8	116
5	Turkey	5	10
6	Greece	4	43
7	Taiwan	4	32
8	England	3	95
9	Italy	3	43
10	Saudi Arabia	3	6

**FIGURE 3:**
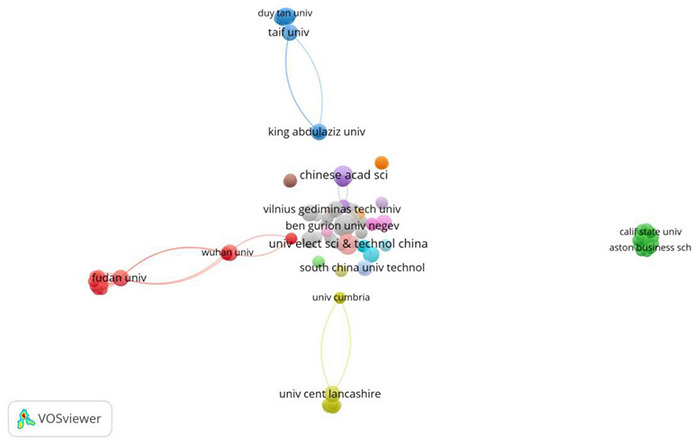
Data distribution of AIEL co-authorship amongst countries from 2010–2021.

Figure 3 comprises 12 nodes and seven wires, where the nodes represent authors and the wires represent cooperative relationships between authors. Almost all the authors who published two articles conducted their studies by cooperating with each other. Meanwhile, this study found that most of the research teams were small in size, and there was almost no cooperation between the groups.

### Roles of Artificial Intelligence in e-Learning

**FIGURE 4:**
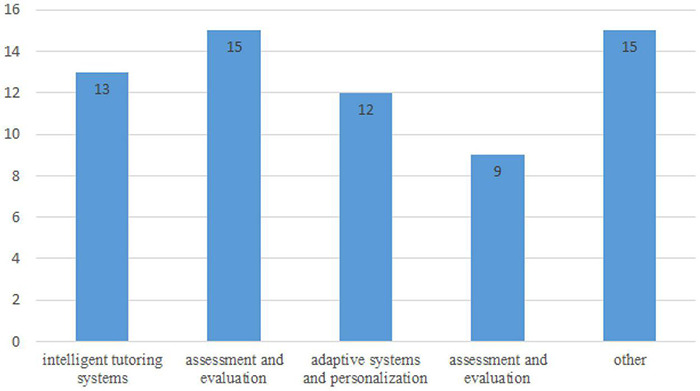
Roles of AIEL research.

This study referred to the subject classification of Hwang and Tu (2021) and Tang et al. (2021) in classifying AIEL, including “adaptive systems and personalization,” “evaluation and assessment,” “profiling and prediction,” “other” and “intelligent tutoring systems.” According to the summarized results, studies between 2010 and 2021 mainly discussed “evaluation and assessment (23.44%)” and “other (23.44%),” followed by “intelligent tutoring systems (20.31%),” “adaptive systems and personalization (18.75%)” and “evaluation and assessment (14.06%)” (see Figure 4). In particular, “Other” mainly analyzed the students’ study behavior intention after constructing AIEL (Miao et al., 2020) or exploring the trends of application and development of AIEL using the interview method (Ally, 2019).

### Keyword Network Analysis

**FIGURE 5:**
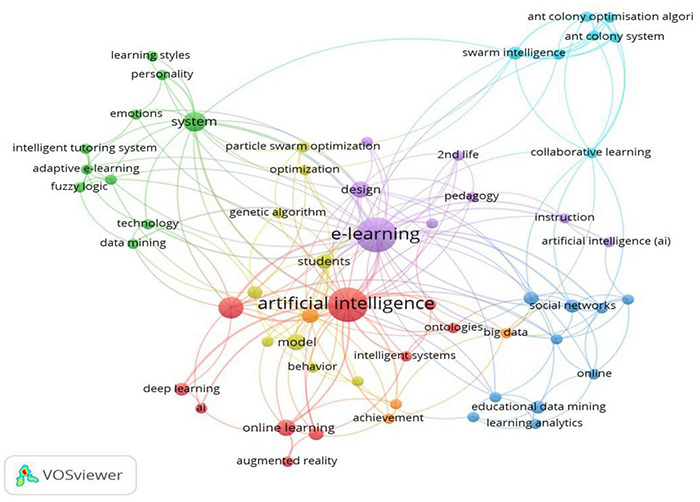
Analysis of keyword co-occurrence from 2010–2021.

Keywords can intuitively reflect the core research content and theme of a discipline. In this paper, the density of the correlation between keywords is used to cluster keywords through a modularity algorithm, and the relevant results are shown in Table 5 and Figure 5.

**TABLE 5:** Cluster terms.

**Clusters**	**Terms**
1 (10 terms)	Artificial intelligence; education; online learning; blended learning; deep learning; augmented reality; expert systems; intelligent systems; ontologies; artificial intelligence
2 (10 terms)	System; adaptive e-learning; data mining; emotions; environment; fuzzy logic; intelligent tutoring system; learning styles; personality; technology
3 (9 terms)	Educational data mining; social networks; systems; computer programming; educational games; learning analytics; management; online; tutoring systems
4 (9 terms)	Model; students; knowledge; behavior; engagement; genetic algorithm; intelligent tutoring systems; optimization; particle swarm optimization
5 (7 terms)	e-Learning; design; 2nd life; instruction; pedagogy; SLOODLE; teaching model
6 (7 terms)	Swarm intelligence; ant colony optimization algorithm; ant colony system; collaborative learning; learners’ behavior; learning objects; learning paths
7 (4 terms)	Performance; achievement; analytics; big data

Cluster 1 reflects the hot vocabularies in the field of learning systems—including artificial intelligence, education, online learning, blended learning, deep learning, augmented reality, expert systems, intelligent systems and ontologies—showing that the papers in Cluster 1 focus on the use of computers and artificial intelligence, the development and building of more sophisticated learning systems and the use of new technologies in the teaching environment. For example, augmented reality technology is used to combine online and offline teaching to facilitate access to more comprehensive learning materials or enhance the teaching and learning experience (Ally, 2019). Meanwhile, Aiyuan and Hui (2021) adopted artificial intelligence in online English learning and used an improved network analysis method to evaluate the time and model of students’ online learning, thus realizing effective application in actual intelligent teaching. Therefore, under the environmental circumstance of combining artificial intelligence and teaching, it is reasonable to use new technologies to improve teaching, and such use is a vital part of enhancing teaching efficiency.

Cluster 2 reflects the hot vocabularies in the field of performance evaluation—including system, adaptive e-learning, data mining, emotions, environment, fuzzy logic, intelligent tutoring systems, learning styles, personality and technology—showing that the literature uses artificial intelligence technology to evaluate students’ learning by taking advantage of students’ emotions and other personality factors to make the online learning system more adaptive (Alsobhi and Alyoubi, 2019). The conclusion drawn from Cluster 2 is that using artificial intelligence to evaluate learning and employing adaptive e-learning can improve students’ learning efficiency and make online learning more helpful.

Cluster 3 reflects the hot vocabularies in the field of artificial intelligence combined with online education—including educational data mining, social networks, systems, computer programming, educational games, learning analytics, management, online and tutoring systems. Amongst them, educational data mining, systems, educational games, and tutoring systems reflect that the core research orientation in the field of education is to focus on the construction of a complete educational system. Through educational data mining (Santos and Boticario, 2015; Yang et al., 2021), learning analytics (Franzoni et al., 2020) and other data collection and analysis methods, the behavioral patterns and emotional differences of personalized students can be analyzed to improve the management ability of the system. Based on suggestions in the literature on the multi-dimensional and overall research direction, social networks can be supported by computer programming and educational artificial intelligence games to promote the development of artificial intelligence applications in online education (Kuk et al., 2017; Petit et al., 2018). Therefore, the combination of educational games and artificial intelligence is a vital approach for studies on using artificial intelligence in teaching.

Cluster 4 reflects the hot vocabularies in the field of artificial intelligence combined with online education—including model, students, knowledge, behavior, engagement, genetic algorithm, intelligent tutoring system optimization and particle swarm optimization. Amongst them, students, knowledge, behavior and engagement focus more on the subject in the process of education system construction. It includes but is not limited to behavioral patterns, teaching engagement and knowledge. In addition, the concept of artificial intelligence is strengthened and highlighted, and the concept of personalized customization is developed and extended. Particularly at the level of artificial intelligence technology, the comprehensive application of various models, genetic algorithms, optimization, particle swarm optimization and other technologies advance the development of education systems in a more intelligent direction (Chang and Ke, 2013; Kuk et al., 2017) and change “tutoring systems” in Cluster 4 to “intelligent tutoring systems.” This indicates that improving the use of intelligent tutoring systems through algorithmic improvements in teaching and research is another important approach in the sustainable development of artificial intelligence in the future.

Cluster 5 reflects the hot vocabularies in a certain theme—including e-learning, design, second life, instruction, pedagogy, SLOODLE and teaching model—showing that the application of the literature focuses on the development of an intelligent learning environment and proposes the creation of a learning environment in Second Life or OpenSimulator, combined with the Moodle learning management system (Crespo et al., 2013). However, an interesting issue is raised in the study approach of Cluster 5, which is that studies in that period tended to enhance students’ learning efficiency by combining simulated environments with LMSs. Those studies are the early research on using artificial intelligence in teaching, and since 2015, relevant studies have either slowed down or disappeared, possibly due to the study approaches and trends on the use of artificial intelligence.

Cluster 6 reflects the hot vocabularies in a certain theme—including swarm intelligence, ant colony optimization, algorithm, ant colony system, collaborative learning, learners’ speech classes like behavior, learning objects and learning paths—which mainly reflect new methods of recommending appropriate learning paths for different learner groups. Through ant colony optimization, ant colony system and other algorithms, we can monitor and improve e-learning modules and courses according to the behaviors of learners (Kurilovas et al., 2014). The conclusion drawn from Cluster 6 shows that algorithmic improvements could enable effective recommendations of learning paths. Furthermore, the studies on this topic were conducted around 2015, after which learning recommendation systems was no longer the topic of relevant studies; thus, studies in this area slowed down.

Cluster 7 reflects the hot vocabularies in a certain theme—including performance, achievement, analytics and big data—which mainly discuss the application of teaching to improve the traditional algorithm, propose a new improved model and develop automatic methods to detect patterns in a large amount of educational data and estimate unknown information and behaviors about students (Kose and Arslan, 2016; Miao et al., 2020). In particular, the study in Cluster 7 mainly analyzed students’ learning results and efficiency through an intelligent e-learning system and verified the study result through statistical analysis.

### Keyword Evolution Analysis

**FIGURE 6:**
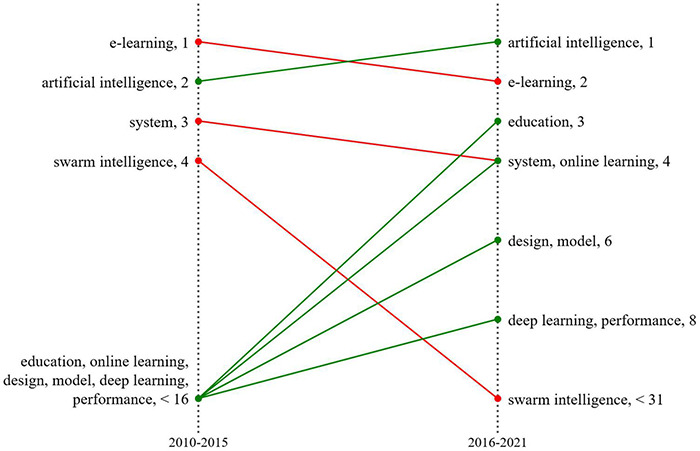
Keyword evolution analysis.

The evolution of AIEL is shown in Figure 6. From 2010 to 2015, the application of AI mainly focused on recommending suitable learning paths for learners and improving their learning benefits through intelligent learning (Kurilovas et al., 2015). However, research from 2016 to 2021 focused on developing new technological applications to assess the learning status of individual students and providing them with helpful learning methods (e.g., statistical learning, data mining and decision trees) (Samarakou et al., 2018; Kavitha and Lohani, 2019; Franzoni et al., 2020). Conversely, compared to recommended applications before 2015, the research in 2016 also focused on applying artificial intelligence technology to integrate teaching and adopted optimized methods to improve the teaching methods of online learning to assist students in learning, as well as to evaluate and optimize learning effects (Barlybayev et al., 2020; Xie and Mai, 2021).

## Discussion and Conclusion

The study analyzed 64 articles on AIEL published in the WOS database between 2010 and 2021. Several studies have shown that the use of artificial intelligence technology has great potential in promoting students’ learning performances and higher-level thinking (Voskoglou and Salem, 2020). In addition, using artificial intelligence technology to diagnose students’ learning problems can not only provide immediate feedback to individual students but also provide information to help teachers improve learning designs (Hung et al., 2014; Bywater et al., 2019). From the analysis results, the conclusions and impacts are as follows:

Most AIEL studies were published in Expert Systems with Applications, Computers in Human Behavior, IEEE Access, and Journal of Intelligent and Fuzzy Systems. Before 2016, the topics mainly focused on the use of a recommendation system to evaluate students’ learning effects to further propose the design of new online learning course modules Kurilovas et al., 2014, 2015; Stantchev et al., 2015). However, since 2017, the research focus has been on the influence of artificial intelligence applications on teaching status (Jalal and Mahmood, 2019; Kavitha and Lohani, 2019; Franzoni et al., 2020). In addition, the top three most-cited journals (co-citation analysis) are Expert Systems with Applications, Computers in Human Behavior, and Journal of Network and Computer Applications. In other words, more researchers in education and educational technology are engaged in AIEL research, especially in the application of artificial intelligence technology in the teaching environment.

From the results of cluster analysis on user keywords, the AIEL cluster analysis showed that the three most important clusters are “behavioral analysis of artificial intelligence combined with online education,” “learning effect evaluation,” and “using artificial intelligence technology to develop and build a perfect learning system.” Furthermore, intelligent systems to assess student learning (Barlybayev et al., 2020; Aiyuan and Hui, 2021; Yang et al., 2021) may be a good reference direction for future research.

The most common roles of artificial intelligence in online learning are “e-learning” and “adaptive e-learning,” followed by “educational data mining” and “engagement and behavior,” which is consistent with the findings on research issues—that is, evaluating student performance through artificial intelligence is the main focus of the AIEL research. In addition, the research on evaluating students’ learning problems to provide them with immediate support, improve their learning effectiveness and performance, and conduct an effective evaluation of follow-up learning is relatively rare. Therefore, it should also be the direction of future research topics.

Most studies employ traditional routine learning methods or interviews with experts in this field, while modern artificial intelligence methods, such as deep learning, are rarely adopted. This may be because these AIEL studies have focused on developing new technology applications to assess the learning status of individual students and help them. This goal is associated with the characteristics of traditional routine learning methods (e.g., statistical learning, recommendation systems, data mining, and decision trees) and knowledge acquisition methods by interviewing experts in the field. That is, knowledge in the field is explicitly expressed and used to make decisions or predictions (Samarakou et al., 2018; Kavitha and Lohani, 2019; Franzoni et al., 2020).

Most AIEL studies have investigated the application of artificial intelligence technologies to integrate teaching and the use of optimized methods to improve the teaching methods of online learning to assist students in learning, as well as evaluate and optimize learning outcomes (Barlybayev et al., 2020; Xie and Mai, 2021). However, there is limited discussion on the rationality and development of artificial intelligence to accumulate skills. Thus, it should also be the direction of future teaching research.

Finally, regarding the research topic, studies on the AIEL field are growing. Most researchers have focused on five directions: adaptive systems and personalization, profiling and prediction, evaluation and assessment, other and intelligent tutoring systems. The result also indicated that research on AIEL is still in its growth period, thus needing more interdisciplinary research. Subsequent research could emphasize the analysis of students’ behaviors, an evaluation of the study’s effectiveness, the technological application of artificial intelligence combined with online learning, and the integration of cross-technology applications (e.g., the AR/VR-integrated teaching modes, the STEAM teaching mode, etc.).

This study has some limitations: the presented keyword nodes can only be presented qualitatively. From the quantitative perspective, the value represented by the node comes from the number of occurrences of the keywords in the entire literature data set. If the software can weigh the number of occurrences of the keyword and the author’s publication order in the specific literature, the study will have a stronger persuasion.

## Data Availability Statement

The original contributions presented in the study are included in the article/supplementary material, further inquiries can be directed to the corresponding author/s.

## Author Contributions

KJ and YL designed the research and provided guidance throughout the entire research process. C-LL and ZC collected the references, did the literature analysis, and wrote the manuscript. XJ and PW helped with translation and offered modification suggestions. TC participated in the literature collection, analysis, and organization. All authors listed have made a substantial, direct, and intellectual contribution to the work, and approved it for publication.

## Conflict of Interest

The authors declare that the research was conducted in the absence of any commercial or financial relationships that could be construed as a potential conflict of interest.

## Publisher’s Note

All claims expressed in this article are solely those of the authors and do not necessarily represent those of their affiliated organizations, or those of the publisher, the editors and the reviewers. Any product that may be evaluated in this article, or claim that may be made by its manufacturer, is not guaranteed or endorsed by the publisher.
